# Mammostrat^® ^as a tool to stratify breast cancer patients at risk of recurrence during endocrine therapy

**DOI:** 10.1186/bcr2604

**Published:** 2010-07-08

**Authors:** John MS Bartlett, Jeremy Thomas, Douglas T Ross, Robert S Seitz, Brian Z Ring, Rodney A Beck, Hans Christian Pedersen, Alison Munro, Ian H Kunkler, Fiona M Campbell, Wilma Jack, Gillian R Kerr, Laura Johnstone, David A Cameron, Udi Chetty

**Affiliations:** 1 Endocrine Cancer Group, Edinburgh Cancer Research Centre, Edinburgh University, Carrington Crescent, Edinburgh EH4 2XU, UK; 2 Edinburgh Cancer Centre, Western General Hospital, Crewe Road South, Edinburgh EH4 2XU, UK; 3 Clarient Inc., 31 Columbia, Aliso Viejo, CA 92656, USA

## Abstract

**Introduction:**

Patients with early-stage breast cancer, treated with endocrine therapy, have approximately 90% 5-year disease-free survival. However, for patients at higher risk of relapse despite endocrine therapy, additional adjuvant therapy, such as chemotherapy, may be indicated. The challenge is to prospectively identify such patients. The Mammostrat^® ^test uses five immunohistochemical markers to stratify patients on tamoxifen therapy into risk groups to inform treatment decisions. We tested the efficacy of this panel in a mixed population of cases treated in a single center with breast-conserving surgery and long-term follow-up.

**Methods:**

Tissue microarrays from a consecutive series (1981 to 1998) of 1,812 women managed by wide local excision and postoperative radiotherapy were collected following appropriate ethical review. Of 1,390 cases stained, 197 received no adjuvant hormonal or chemotherapy, 1,044 received tamoxifen only, and 149 received a combination of hormonal therapy and chemotherapy. Median age at diagnosis was 57, 71% were postmenopausal, 23.9% were node-positive and median tumor size was 1.5 cm. Samples were stained using triplicate 0.6 mm^2 ^tissue microarray cores, and positivity for p53, HTF9C, CEACAM5, NDRG1 and SLC7A5 was assessed. Each case was assigned a Mammostrat^® ^risk score, and distant recurrence-free survival (DRFS), relapse-free survival (RFS) and overall survival (OS) were analyzed by marker positivity and risk score.

**Results:**

Increased Mammostrat^® ^scores were significantly associated with reduced DRFS, RFS and OS in estrogen receptor (ER)-positive breast cancer (*P *< 0.00001). In multivariate analyses the risk score was independent of conventional risk factors for DRFS, RFS and OS (*P *< 0.05). In node-negative, tamoxifen-treated patients, 10-year recurrence rates were 7.6 ± 1.5% in the low-risk group versus 20.0 ± 4.4% in the high-risk group. Further, exploratory analyses revealed associations with outcome in both ER-negative and untreated patients.

**Conclusions:**

This is the fifth independent study providing evidence that Mammostrat^® ^can act as an independent prognostic tool for ER-positive, tamoxifen-treated breast cancer. In addition, this study revealed for the first time a possible association with outcome regardless of node status and ER-negative tumors. When viewed in the context of previous results, these data provide further support for this antibody panel as an aid to patient management in early-stage breast cancer.

## Introduction

Endocrine therapy is a highly effective adjuvant therapy for estrogen receptor (ER)-positive early breast cancer [1-3]. However, critical reviews of the early tamoxifen trials, with extensive follow-up [1], demonstrate that many patients derive little or no lasting benefit from adjuvant tamoxifen therapy [1]. Evidence from over 40,000 women randomized between no adjuvant therapy and 5 years of tamoxifen demonstrated that > 50% of women with node-negative breast cancer receiving no adjuvant treatment were disease free 15 years later. Adjuvant tamoxifen prevented relapse in < 14% of patients whilst > 34% experienced disease relapse (almost half whilst receiving tamoxifen therapy [1]).

The recent development of third-generation aromatase inhibitors has shown statistically significant and clinically useful improvements in outcome, versus tamoxifen, in ER-positive breast cancers. However, the proportion of patients deriving added benefit is small, particularly in the first 2 to 3 years of treatment [4]. A recent overview of two trials comparing aromatase inhibitors against tamoxifen (Arimidex, Tamoxifen, Alone or in Combination Trial and Breast International Group-1-98) suggests that during the first 2 years of treatment with aromatase inhibitors only one recurrence was prevented for every 100 to 150 patients treated [5,6].

It is now widely acknowledged that ER-expressing breast cancer is biologically heterogeneous and that this complexity accounts for part of the variation in clinical outcome [7]. The challenge is to exploit this knowledge to optimize adjuvant therapy for early ER-positive breast cancers. To date, clinicians have relied upon clinical and pathologic factors to predict outcome and select adjuvant therapy. Combinations of risk factors such as the Nottingham Prognostic Index [8] and Adjuvant Online! [9] are useful for risk-stratifying patients, especially when patients fall into either high-risk or low-risk strata. Documented variation in outcome for patients with similar risk profiles, however, makes it clear that there is considerable clinical diversity not accounted for by these classifiers.

More recently, the advent of genome-wide cDNA microarrays revealed that tumor specimens could be classified according to gene expression profiles [10-12]. This information has begun to be translated into clinical assays to predict outcome and response to therapy. Two such assays relying upon measurement of gene expression in tumor RNA - OncotypeDx and Mammaprint [13,14] - are currently being evaluated in prospective clinical trials. These molecular assays in large part measure the same biology assessed by conventional pathologic tools; for example, proliferation, grade (not independent from proliferation), and hormone receptor status. These assays are relatively high cost, technically demanding (to the extent they are performed only in single centers at present), and therefore potentially limited in their ultimate utility.

An alternative approach has been utilized to develop the immunohistochemical (IHC) assay Mammostrat^®^, with potential for implementation in routine pathology assessment of breast cancers. Combining the novel information that has emerged from gene expression profiling experiments with conventional IHC technology led to the design of a five-biomarker assay measuring SLC7A5, HTF9C, P53, NDRG1, and CEACAM5. These markers are independent of one another and do not directly measure either proliferation or hormone receptor status, and whilst there are caveats regarding the extrapolation of immunohistochemistry to determine the mutational status of individual genes, as a combined index of five markers this panel has previously been evaluated in three independent institutional cohorts, and in a fourth combined analysis with the clinical trial specimens available from the NSABP 14 and B20 adjuvant clinical trials [15,16]. In principle, therefore, Mammostrat^® ^results could be interpreted in conjunction with conventional histopathological information about the proliferation and hormone receptor status of a tumor. In the present article, we report the result of the largest institutional validation study performed to date. We explored the relationship of Mammostrat^® ^risk stratification in risk classes identified by standard pathologic and clinical prognostic variables.

## Materials and methods

The Edinburgh Breast Conservation Series represents a fully documented, consecutive cohort of 1,812 patients treated by breast-conservation surgery, axillary node sampling or clearance, and whole-breast radiotherapy at the Edinburgh Cancer Centre between 1981 and 1998. Over this period patients were managed by a specialist multidisciplinary team including surgeons, radiologists, pathologists, and oncologists. Eligible patients were those considered suitable for breast-conserving therapy and were T1 or T2 (< 3 cm), N0 or N1, and M0 on conventional TNM staging. Postoperative breast radiotherapy was given over 4 to 5 weeks at a dose of 45 Gy in 20 to 25 fractions. Data are available on adjuvant treatment, tumor size, ER status, lymph node status and outcome with a minimum follow-up of 9 years.

Following ethical approval (Lothian Local Research Ethics 04), tissue blocks were retrieved from all cases, and sufficient material was available from 1,686 cases for assembly into tissue microarrays; all cases with available tissue were regarded by a single pathologist [17]. The ethical review board confirmed that informed consent was not required for this study.

For the current study, patients treated with chemotherapy only (*n *= 146) were excluded and 1,540 cases were stained using the Mammostrat^® ^antibody panel (Table 1). These cases included tumors treated with adjuvant tamoxifen without chemotherapy (1,102 cases), with other hormonal therapy (92 cases), and with both hormone therapy and chemotherapy (149 cases). In addition, 197 cases received no adjuvant hormone therapy or chemotherapy.

**Table 1:** Patient characteristics within the study population by subgroup

Parameter	All cases (*n *= 1,540)	All ER-positive (*n *= 1,189)	**ER-positive tamoxifen only**^**a **^**(*n *= 831)**	**ER-positive, node-negative, tamoxifen only**^**a **^**(*n *= 657)**
Age				
< 50 years	660 (42.8%)	505 (42.5%)	284 (34.2%)	243 (37.0%)
> 50 years	879 (57.1%)	683 (57.4%)	547 (65.8%)	414 (63.0%)
Missing	1 (0.1%)	1 (0.1%)		
Grade				
1	411 (26.7%)	359 (30.2%)	269 (32.4%)	215 (32.7%)
2	710 (46.1%)	581 (48.9%)	416 (50.1%)	323 (49.2%)
3	381 (24.7%)	233 (19.6%)	135 (16.2%)	109 (16.6%)
Missing	38 (2.5%)	16 (1.3%)	11 (1.3%)	10 (1.5%)
Nodes				
Negative	1164 (75.6%)	889 (74.8%)	657 (79.1%)	657 (100%)
1 to 3	321 (20.8%)	264 (22.2%)	154 (18.5%)	
4 to 9	44 (2.9%)	29 (2.4%)	17 (2.0%)	
10+	9 (0.6%)	5 (0.4%)	3 (0.4%)	
Missing	2 (0.1%)	2 (0.2%)		
Size				
< 20 mm	1150 (74.7%)	903 (75.9%)	648 (78.0%)	531 (80.8%)
> 20 mm	314 (20.4%)	224 (18.8%)	148 (17.8%)	99 (15.1%)
Missing	76 (4.9%)	62 (5.2%)	35 (4.2%)	27 (4.1%)
Estrogen receptor				
< 2	278 (18.1%)	19 (1.6%)	15 (1.8%)	14 (2.1%)
Allred 3 to 5	347 (22.5%)	347 (29.2%)	213 (25.6%)	174 (26.5%)
Allred 6 to 8	823 (53.4%)	823 (69.2%)	603 (72.6%)	469 (71.4%)
Missing	92 (6.0%)			
Progesterone receptor				
< 2	242 (15.7%)	131 (11.0%)	90 (10.8%)	74 (11.3%)
Allred 3 to 5	334 (21.7%)	245 (20.6%)	180 (21.6%)	144 (21.9%)
Allred 6 to 8	866 (56.2%)	785 (66.0%)	542 (65.2%)	422 (64.2%)
Missing	98 (6.4%)	28 (2.4%)	19 (2.3%)	17 (2.6%)

ER, estrogen receptor. ^a^Tamoxifen only, patients treated with adjuvant tamoxifen without adjuvant chemotherapy.

### Biomarker analysis

Samples were stained, using triplicate 0.6 mm^2 ^tissue microarray cores, and positivity for p53, HTF9C (recently re-named TRIMT2A), CEACAM5, NDRG1, and SLC7A5 was recorded as previously described [15,16]. Briefly, scoring was on a semiquantitative scale, where invasive breast cancer epithelium present in each tissue core was scored as negative (0), weak (1), or strong (2). CEACAM5 and NDRG1 were scored for cytoplasmic or membrane staining, SLC7A5 for membrane staining, HTF9C (TRIMT2A) for cytoplasmic staining, and p53 for nuclear staining. With the exception of NDRG1, cases were considered positive if staining was present on > 10% of invasive tumor cells. NDRG1 was scored as positive only when a core exhibited homogeneous staining across the available sample.

A single investigator scored all replicates and then inconsistencies between replicates were reviewed by two investigators using an online image database to resolve inconsistent replicates. The pair-wise concordance between replicates prior to review was 95% for CEACAM5, 88% for p53, 95% for SLC7A5, 89% for HTF9C (TRIMT2A), and 93% for NDRG1. Consensus scores across triplicate cores were generated considering the case as positive if any of the three replicates was scored positive. A Mammostrat^® ^risk score was generated by combining the five staining results as either positive or negative according to the prospectively specified algorithm [15]. Staining results were established prior to transfer to the statistics team for cross-reference to the clinical outcome data and analysis.

### Statistical analysis

SPSS version 14.0 (SPSS Inc., Chicago, IL, USA) was used for all statistical analysis. Kaplan-Meier and log-rank analysis were used to compare relapse-free survival (RFS), distant recurrence-free survival (DRFS), and overall survival (OS). Hazard ratios and their confidence intervals were calculated from log-rank statistics. All reported *P *values are two-sided. In accordance with the statistical analysis plan, patients were stratified into low, medium and high risk, based on the integrated biomarker expression profile (risk index).

Two prospectively defined primary analyses were performed to address the *a priori *hypothesis that Mammostrat^® ^is a prognostic tool for ER-positive tamoxifen-treated breast cancer. Firstly, DRFS was determined in ER-positive, node-negative cases treated with tamoxifen only and in ER-positive, tamoxifen-treated cases irrespective of nodal status. Further, exploratory, analyses are reported in untreated cases, ER-negative cases, all ER-positive cases, and all cases irrespective of hormonal status and treatment. Univariate and multivariate analyses were performed for the Mammostrat^® ^score in each of these analyses with respect to RFS, DRFS and OS (breast cancer specific).

## Results

Of the 1,540 patients included in this study, 25% were grade 3, 24% were node positive, 20% had large tumors (> 2.0 cm) and 18% were ER-negative (Allred < 3) (see Table 1 for clinicopathological parameters). For the primary, prospectively defined, analyses in this study, we included only ER-positive tumors treated with tamoxifen, excluding patients treated with adjuvant chemotherapy (all patients received adjuvant radiotherapy), and then stratified by nodal status. The 831 ER-positive patients treated only with adjuvant tamoxifen were predominantly node-negative (79%, 657/831), presented with tumor size < 2 cm (78%) and were ER rich (72% Allred 6 or greater) (Table 1). For this population, 66% were > 50 years of age and were regarded as postmenopausal (Table 1).

Across all patients, Mammostrat^® ^assigned 46.6% of tumors to low-risk, 19.8% to moderate-risk and 18.1% to high-risk strata (15.6% of cases had missing data for one or more marker; complete data were available for 1,300 tumors) (Table 2). In ER-expressing, node-negative patients treated with tamoxifen, 51.9% were low risk, 21.2% moderate risk, and 13.4% high risk (*n *= 568, 13.5% missing data) (Table 2). Significantly more cases were assigned to the Mammostrat^® ^high-risk group in the ER-negative population versus the ER-positive population (45% vs. 16%, respectively; *P *< 0.0001). No marked differences in Mammostrat^® ^scores were observed between the ER-positive groups (all patients, tamoxifen-treated cases, and tamoxifen-treated, node-negative cases) (Table 2).

**Table 2:** Biomarker results by subgroup

Biomarker	All cases	All ER-positive	**ER-positive tamoxifen only**^**a**^	**ER-positive, node-negative, tamoxifen only**^**a**^
SLC7A5				
0	1231 (79.9%)	1035 (87.0%)	732 (88.1%)	578 (88.0%)
1	139 (9.0%)	77 (6.5%)	51 (6.1%)	38 (5.8%)
2	41 (2.7%)	24 (2.0%)	11 (1.3%)	9 (1.4%)
Missing^b^	129 (8.4%)	53 (4.5%)	37 (4.5%)	32 (4.9%)
HTF9C				
0	1234 (80.1%)	1015 (85.4%)	712 (85.7%)	559 (85.1%)
1	103 (6.7%)	69 (5.8%)	43 (5.2%)	35 (5.3%)
2	72 (4.7%)	46 (3.9%)	36 (4.3%)	25 (3.8%)
Missing	131 (8.5%)	59 (5.0%)	40 (4.8%)	38 (5.8%)
NDRG1				
0	1085 (70.5%)	904 (76.0%)	624 (75.1%)	495 (75.3%)
1	175 (11.4%)	127 (10.7%)	96 (11.6%)	75 (11.4%)
2	148 (9.6%)	100 (8.4%)	66 (7.9%)	47 (7.2%)
Missing	132 (8.6%)	58 (4.9%)	45 (5.4%)	40 (6.1%)
CEACAM5				
0	1251 (81.2%)	1005 (84.5%)	699 (84.1%)	550 (83.7%)
1	75 (4.9%)	67 (5.6%)	46 (5.5%)	39 (5.9%)
2	77 (5.0%)	61 (5.1%)	44 (5.1%)	29 (4.4%)
Missing	137 (8.9%)	56 (4.7%)	44 (5.3%)	39 (5.9%)
P53				
0	1040 (67.5%)	884 (74.3%)	619 (74.5%)	479 (72.9%)
1	229 (14.9%)	166 (14.0%)	119 (14.3%)	101 (15.4%)
2	117 (7.6%)	67 (5.6%)	39 (4.7%)	30 (4.6%)
Missing	154 (10.0%)	72 (6.1%)	54 (6.5%)	47 (7.2%)
LMH				
Low	717 (46.6%)	643 (54.1%)	444 (53.4%)	341 (51.9%)
Medium	305 (19.8%)	244 (20.5%)	175 (21.1%)	139 (21.2%)
High	278 (18.1%)	168 (14.1%)	112 (13.5%)	88 (13.4%)
Missing	240 (15.6%)	134 (11.3%)	100 (12.0%)	89 (13.5%)
Total	1300	1055	731	568

ER, estrogen receptor; LMH, low, medium, or high. ^a^Tamoxifen only, patients treated with adjuvant tamoxifen without adjuvant chemotherapy. ^b^Missing data due to loss of cores from tumor microarrays or no tumor in the tumor microarray core.

### ER-positive, node-negative, tamoxifen-treated cases

**Figure 1:**
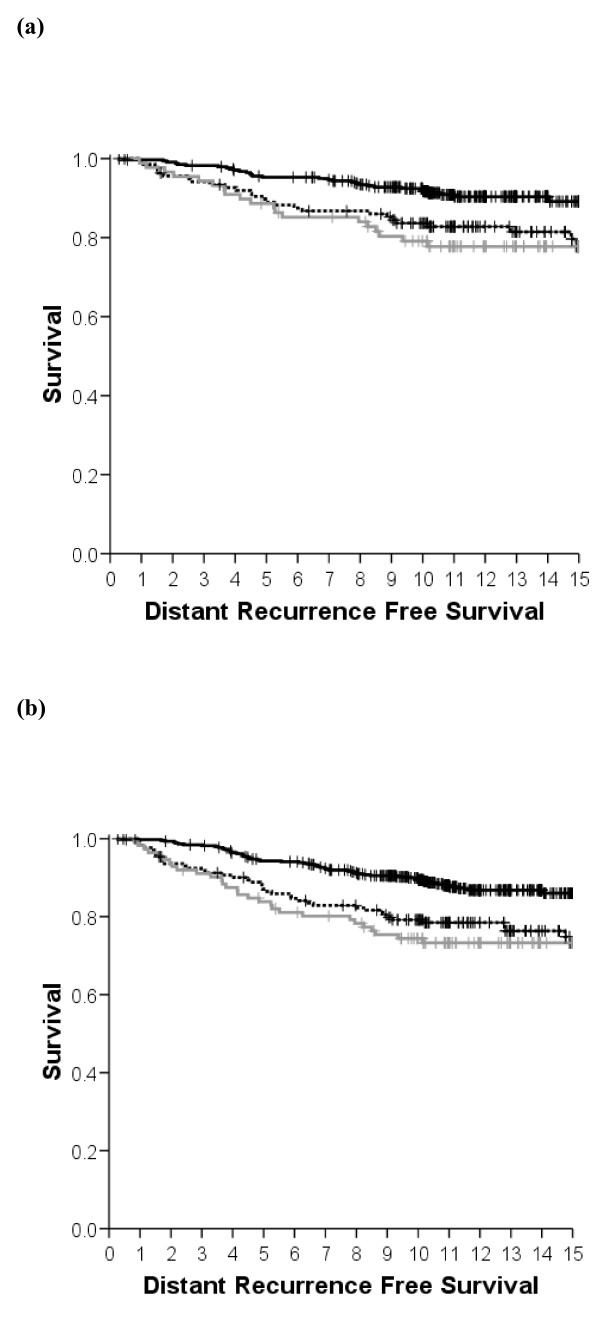
**Co-primary endpoints**. Kaplan-Meier distant recurrence-free survival (DRFS) survival curves for breast-conserving surgery cases. Solid lines, Mammostrat^® ^score low risk; dotted lines, Mammostrat^® ^score medium risk; grey lines, Mammostrat^® ^score high risk. **(a) **Estrogen receptor (ER)-positive, node-negative breast cancers treated with adjuvant tamoxifen only. **(b) **ER-positive, tamoxifen-treated breast cancers (any nodal status).

Following univariate analysis of the subgroup of ER-positive, node-negative tumors treated with tamoxifen (without chemotherapy, *n *= 568; Table 2), a significant association was observed between the Mammostrat^® ^risk score and RFS (*P *= 0.016) and between the risk score and DRFS (*P *= 0.003), with a trend observed for overall survival (Table 3). At 10 years, the low-risk group had a 7.6% (standard error = 1.5%) distant recurrence rate, the moderate-risk group 16.3% (standard error = 3.2%), while the high-risk group exhibited a 20.9% distant recurrence rate (Figure 1a, standard error = 4.4%, *n *= 88). The overall distant recurrence rate for the unstratified population was 11.1% (standard error = 1.2%). Similar statistically significant differences were observed for RFS (Figure S1A in Additional file 1) and a trend was observed for OS (data not shown). Tumors with both high and moderate Mammostrat^® ^risk scores showed increased relative risk of local and distant relapse during follow-up continuing over 15 years (Figure 1a).

**Table 3:** Results of univariate analysis for individual biomarkers and Mammostrat^® ^risk score

	Recurrence-free survival	Distant recurrence-free survival	Overall survival
ER-positive, tamoxifen treated, node-negative			
SLC7A5	NS	NS	NS
HTF9C	NS	NS	NS
NRDG1	NS	1.79 (1.11 to 2.88), *P *= 0.015	NS
CEACAM5	NS	NS	NS
P53	NS	1.60 (1.01 to 2.54), *P *= 0.045	NS
LMH	*P *= 0.016	*P *= 0.003	NS
LvH	1.78 (1.08 to 2.95), *P *= 0.025	2.30 (1.31 to 4.05), *P *= 0.004	
LvM	1.73 (1.12 to 2.68), *P *= 0.014	2.01 (1.21 to 3.35), *P *= 0.007	
ER-positive, tamoxifen treated			
SLC7A5	NS	NS	NS
HTF9C	1.61 (1.04 to 2.50), *P *= 0.032	1.87 (1.17 to 2.98), *P *= 0.008	NS
NRDG1	1.45 (1.02 to 2.05), *P *= 0.037	1.83 (1.26 to 2.67), *P *= 0.001	1.64 (1.21 to 2.21), *P *< 0.0005
CEACAM5	NS	NS	NS
P53	1.43 (1.02 to 2.01), *P *= 0.036	1.64 (1.13 to 2.39), *P *= 0.008	NS
LMH	*P *= 0.003	*P *= 0.0001	*P *= 0.006
LvH	1.80 (1.20 to 2.70), *P *= 0.005	2.23 (1.43 to 3.49), *P *= 0.0005	1.99 (1.17 to 3.41), *P *= 0.012
LvM	1.65 (1.15 to 2.35), *P *= 0.006	1.99 (1.33 to 2.97), *P *= 0.0008	1.88 (1.20 to 2.96), *P *= 0.006
All ER-positive cases			
SLC7A5	NS	NS	NS
HTF9C	NS	1.51 (1.01 to 2.27), *P *= 0.048	NS
NRDG1	1.51 (1.13 to 2.02), *P *= 0.005	1.84 (1.34 to 2.52), *P *= 0.0001	1.82 (1.42 to 2.34), *P *< 0.0005
CEACAM5	NS	NS	NS
P53	1.39 (1.05 to 1.85), *P *= 0.02	1.56 (1.14 to 2.13), *P *= 0.005	NS
LMH	*P *< 0.0001	*P *< 0.00001	*P *< 0.00001
LvH	1.80 (1.26 to 2.57)	2.19 (1.49 to 3.24), *P *= 0.00008	2.16 (1.39 to 3.56), *P *= 0.0006
LvM	1.78 (1.31 to 2.40), *P *= 0.0002	2.10 (1.49 to 2.95), *P *= 0.00002	2.26 (1.55 to 3.30), *P *= 0.00002
All cases			
SLC7A5	1.45 (1.08 to 1.95), *P *= 0.012	1.63 (1.19 to 2.22), *P *= 0.002	1.39 (1.06 to 1.80), *P *= 0.015
HTF9C	1.45 (1.07 to 1.95), *P *= 0.015	1.57 (1.14 to 2.16), *P *= 0.005	1.37 (1.05 to 1.79), *P *= 0.021
NRDG1	1.58 (1.25 to 2.01), *P *< 0.0005	1.86 (1.43 to 2.40), *P *< 0.0005	1.76 (1.43 to 2.17), *P *< 0.0005
CEACAM5	NS	1.44 (1.03 to 2.02), *P *= 0.034	NS
P53	1.57 (1.25 to 1.98), *P *< 0.0005	1.80 (1.40 to 2.31), *P *= 0.010	1.32 (1.07 to 1.63), *P *= 0.010
LMH	*P *< 0.00001	*P *< 0.00001	*P *< 0.00001
LvH	2.02 (1.54 to 2.64)	2.53 (1.89 to 3.40)	2.62 (1.89 to 3.63)
LvM	1.74 (1.33 to 2.28)	2.02 (1.49 to 2.73)	2.24 (1.61 to 3.11)

All individual markers analyzed as dichotomous variables. ER, estrogen receptor; LMH, *P *value for significance of Mammostrat^® ^score; LvM, hazard ratio (95% confidence interval) for low-risk versus medium-risk cases classified by Mammostrat^®^; LvH, hazard ratio (95% confidence interval) for low-risk versus high-risk cases classified by Mammostrat^®^; NS, nonsignificant.

In multivariate regression analysis, menopausal status, multifocality, and HER2 were significant predictors of RFS (Table 4), with a trend (*P *= 0.076) towards significance for Mammostrat^® ^scores. Only the Mammostrat^® ^score (*P *= 0.059) and HER2 status (*P *= 0.092) trended towards significance for the prediction of DRFS and no risk factors predicted for OS (Table 4). Exploratory univariate analyses of the individual biomarkers revealed that NRDG1 and p53 were individually significantly associated with reduced DRFS, but only NDRG1 was significantly associated with OS (Table 3).

**Table 4:** Results of multivariate regression analysis including Mammostrat^® ^risk score

	**ER-positive, node-negative, tamoxifen only**^**a**^	**ER-positive, tamoxifen only**^**a**^	All ER-positive cases	All Eligible cases
Relapse-free survival				
Nodal status	*	5.4 × 10^-8^	0.0001	2.8 × 10^-9^
Grade	NS	0.046	NS	NS
Size	NS	0.010	7.0 × 10^-5^	0.0007
Multifocality	0.037	0.001	0.0004	0.0004
Menopausal status	0.031	0.013	0.048	NS
Age	NS	NS	NS	NS
HER2	0.012	0.0005	0.003	0.001
PgR	NS	NS	0.079	NS
ER	*	*	*	NS
Mammostrat^®^	0.076	0.064	0.025	0.0007
Distant recurrence-free survival				
Nodal status	*	3.7 × 10^-7^	5.1 × 10^-5^	4.6 × 10^-9^
Grade	NS	0.067	NS	NS
Size		0.091	0.0004	0.002
Multifocality	NS	NS	0.030	0.082
Menopausal status	NS	NS	NS	NS
Age	NS	NS	NS	NS
HER2	0.092	0.01	0.021	0.024
PgR	NS	NS	NS	NS
ER	*	*	*	NS
Mammostrat^®^	0.059	0.012	0.005	6.6x10-5
Overall survival				
Nodal status	*	2.5 × 10^-6^	6.9 × 10^-5^	9.1 × 10^-9^
Grade	NS	0.074	0.036	0.088
Size		0.018	0.00021	0.011
Multifocality	NS	NS	0.061	NS
Menopausal status	NS	NS	NS	NS
Age	NS	0.080	0.037	0.017
HER2	NS	NS	NS	NS
PgR	NS	0.059	0.085	NS
ER	*	*	*	0.001
Mammostrat^®^	NS	NS	0.0023	0.005

Data represent *P *value for significance of individual factors in multivariate analyses. ER, estrogen receptor; NS, not significant. Nodal status, node-positive versus node-negative; grade, grade 1, grade 2 or grade 3; size, < 2 cm vs. ≥ 2 cm; multifocality, presence or absence of multifocal disease; menopausal status, premenopausal versus postmenopausal; age, < 55 years versus ≥ 55 years; HER2, HER2-positive versus HER2-negative; PgR (progesterone receptor) and ER, hormone receptor-positive versus hormone receptor-negative; Mammostrat^®^, risk category low, medium or high. ^a^Tamoxifen only, patients treated with adjuvant tamoxifen without adjuvant chemotherapy. * = not relevant as no stratification, e.g. nodal status in node negative patients.

### ER-positive, tamoxifen-treated cases (node-negative and node-positive combined)

Analysis of both node-positive and node-negative, tamoxifen-treated, ER-positive cases with complete Mammostrat^® ^results (*n *= 731; Table 2) was also performed. Univariate analysis of these patients showed a significant association between the Mammostrat^® ^risk score and DRFS (*P *= 0.0001; Figure 1b), between the score and RFS (*P *= 0.003; Figure S1B in Additional file 1), and between the score and OS (*P *= 0.001; Table 3, and Figure S2B in Additional file 1). At 10 years, the low-risk group had a 10.1% (standard error = 1.5%) distant recurrence rate, the moderate-risk group 20.8% (standard error = 3.1%), while the high-risk group exhibited a 25.6% distant recurrence rate (Figure 1b, standard error = 4.2%, *n *= 112). The overall distant recurrence rate for the unstratified population was 16.8% (standard error = 1.0%). Exploratory univariate analyses of the individual biomarkers revealed that HTF9C and NRDG1 were individually significantly associated with reduced RFS, DRFS, and OS, whilst p53 was associated with RFS and DRFS only (Table 3).

In multivariate analysis, the Mammostrat^® ^risk score remained an independent predictor of DRFS (*P *= 0.012) along with nodal status and HER2 status (all *P *< 0.05). For OS, the Mammostrat^® ^risk score was also an independent predictor of outcome (*P *= 0.017) with nodal status and HER2 (Table 4). For RFS, the nodal status, tumor size, grade, menopausal status, multifocality and HER2 status were significant risk predictors with a trend towards significance for Mammostrat^® ^scores (*P *= 0.064).

### All ER-positive cases

**Figure 2:**
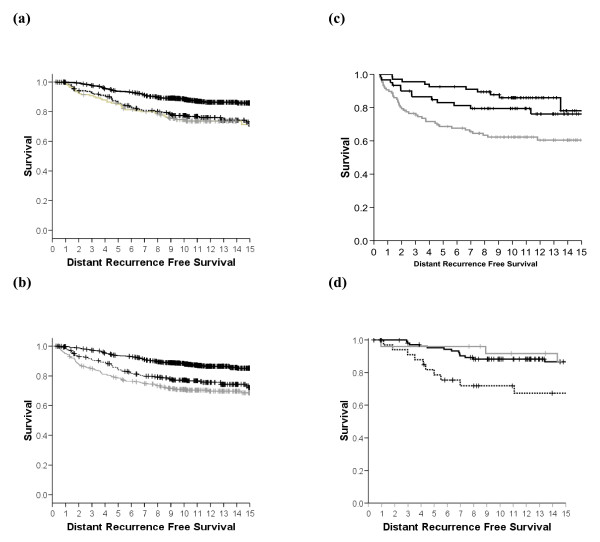
**Exploratory analysis**. Kaplan-Meier distant recurrence-free survival (DRFS) survival cures for breast-conserving surgery cases. Solid lines, Mammostrat^® ^score low risk; dotted lines, Mammostrat^® ^score medium risk; grey lines, Mammostrat^® ^score high risk. **(a) **All estrogen receptor (ER)-positive breast cancers. **(b) **All breast cancers regardless of treatment and hormonal status. **(c) **Untreated breast cancers. **(d) **ER-negative breast cancers.

In univariate analysis of all 1,189 ER-positive cases (of which data for low, medium or high Mammostrat Risk scores were available in 1,055 cases; Table 2), there was a significant association between Mammostrat^® ^score and DRFS (*P *< 0.00001; Figure 2a), between score and RFS (*P *< 0.0001; Figure S1C in Additional file 1), and between score and OS (*P *< 0.00001; Figure S2C in Additional file 1) (Table 3 and Figure 2a). Exploratory univariate analyses of the individual biomarkers revealed that HTF9C, NRDG1, and p53 were individually significantly associated with reduced RFS, DRFS, and OS (Table 3).

In multivariate analyses within this patient group, Mammostrat^® ^scores were independent predictors of RFS (*P *= 0.025 with nodal status, pathological size, multifocality, menopausal status and HER2), of DRFS (*P *= 0.005 with nodal status, pathological size, multifocality and HER2), and of OS (*P *= 0.0023, with pathological size, grade and nodal status also significant).

### All eligible cases (1,300 cases)

Univariate analysis of all cases with complete Mammostrat^® ^data irrespective of ER status, nodal status or treatment showed a highly significant association between Mammostrat^® ^score and DRFS (Figure 2b), between score and RFS (Figure S1D in Additional file 1), and between score and OS (all *P *< 0.00001; Figure S2D in Additional file 1). Exploratory univariate analyses of the individual biomarkers revealed that SLC7A5, HTF9C, NRDG1, and p53 were individually significantly associated with reduced RFS, DRFS, and OS, whilst CEACAM5 was significantly associated only with DRFS (Table 3).

In multivariate analyses, Mammostrat^® ^scores were independent predictors of RFS (*P *= 0.0007, with nodal status, pathological size, multifocality, and HER2 status all *P *< 0.001), DRFS (*P *= 0.00007, with nodal status, pathological size, and HER2 status all *P *< 0.02), and OS (*P *= 0.005 with ER, pathological size and nodal status also significant).

### ER-negative cases and untreated cases

The Breast Conservation Series cohort analyzed included small numbers of ER-negative cases (265 with complete Mammostrat^® ^data) and untreated cases (both ER-positive and ER-negative, 167 with complete Mammostrat^® ^data). In univariate analysis, ER-negative cases showed a significant relationship with DRFS (*P *= 0.009 Figure 2c) while untreated cases also showed a significant outcome (*P *= 0.026 Figure 2d). Similar results were observed for RFS and OS (see Figures S1E, S1F, S2E, and S2F in Additional file 1). Therefore, whilst ER-negative cases were more frequently designated high risk than ER-positive cases (see above), there was no evidence in these exploratory analyses that Mammostrat^® ^performed differently in ER-negative cases and untreated breast cancers when compared with ER-positive cases.

## Discussion

In a retrospective analysis of ER-positive early breast cancer, treated in a single institution with breast-conserving therapy, a prognostic score derived from a simple five-antibody test (Mammostrat^®^) was significantly associated with RFS, DRFS and OS, and was independent of standard clinical and pathological risk factors. In a population-based analysis, including all breast cancers irrespective of ER status, the relationships were preserved (Figure 2b, Figures S1D and S2D in Additional file 1, and Table 4). Subgroup analyses of ER-positive cancers, node-positive versus node-negative cancers, tamoxifen-treated cancers, ER-negative cancers and untreated cancers showed no group in which the Mammostrat^® ^score failed to select patients at high risk and low risk of recurrence (Figures 1 and 2).

In the prospectively defined target population - ER-positive, node-negative patients treated with tamoxifen therapy only (*n *= 568) - low-risk patients had a 10-year distant recurrence rate of 7.6% compared with 20.9% for high-risk patients. Multivariate analysis of this population and of the slightly larger ER-positive, tamoxifen-treated group (with both node-positive and node-negative cancers, *n *= 731) did not, in this low-risk population treated with breast-conserving surgery and radiotherapy, identify Mammostrat^® ^as an independent risk factor. However, in these subpopulations only nodal status was consistently linked to outcome (RFS, DRFS, and OS). It would appear that the small number of events in this subgroup restricts the power to robustly identify key prognostic variables. This interpretation is further supported by the consistent impact of Mammostrat^® ^across all populations in univariate analyses (Table 3), exemplified by the almost identical hazard ratios observed in all subgroups analyzed.

This is the third independent institutional study supporting the association of Mammostrat^® ^with clinical outcome independent of conventional risk factors, and is consistent with results from the study of Mammostrat^® ^in the NSABP B14 and B20 clinical trial samples [15,16]. In the current study, Mammostrat^® ^appears to function as a prognostic tool in node-positive and node-negative disease and in both ER-negative and ER-positive populations, and acts consistently independently of menopausal status. In the published NSABP B20 study, patients deemed high risk had a robust response to adjuvant cytotoxic chemotherapy - suggesting that the test is identifying patients that would benefit from a more aggressive treatment regimen [14].

As with other multiparameter prognostic tools, Mammostrat^® ^appears to identify biological drivers of disease relapse that complement conventional pathological markers (grade, tumor size, nodal status) and other biological markers (for example, HER2). To date, we have analyzed the results of the Mammostrat^® ^panel independently of these biological and clinical risk parameters. Unlike the OncotypeDx and Mammaprint assays, therefore, Mammostrat^® ^does not incorporate hormone receptor status, HER2 status, or measures of proliferation into its risk-stratification algorithm, allowing it to be performed independently of current measurements of growth and hormone receptor status and Ki67 staining or mitotic count indexes. Incorporation of Mammostrat^® ^into nomograms that weight clinical stratifiers and these conventional biomarkers, such as Adjuvant Online! and the Nottingham Prognostic Index, has the potential to give a full accounting of the clinically relevant biologic diversity of breast cancer in considering therapy options. We are currently exploring such an analysis within a sufficiently powered patient cohort.

Breast cancer prognostic markers remain central for treatment decisions and, particularly for ER-positive disease, there is an ongoing debate as to the role of chemotherapy in low-risk breast cancers. There is wide consensus that the currently available prognostic markers do not adequately stratify breast cancer - this is backed up by data from the Oxford overviews [1], which show that a significant minority of patients do not require chemotherapy and that the role of chemotherapy in low-risk, ER-positive breast cancer remains uncertain. Two international trials (TailorX and Mindact) are seeking to explore the value of using additional biological markers to further risk stratify breast cancers (OncotypeDx and Mammaprint, respectively) and to identify patient populations for whom aggressive treatment with chemotherapy is of little or no benefit. We are unaware of any study currently seeking to evaluate different biomarker approaches in a direct comparison.

Current trials rely on complex molecular profiles using either expression arrays or multiplex quantitative PCR techniques that must be performed in a single central laboratory. Neither technique has been shown to be widely applicable in routine diagnostic pathology. Immunohistochemistry, however, has wide application and, with appropriate external quality assurance (for example, NEQAS UK), is highly consistent across multiple laboratories. Whilst, to date, the Mammostrat^® ^IHC profile has only been performed in a central laboratory, there is evidence that this technology will prove applicable in routine diagnostic pathology. The adaptation of these methods to merge with current developments in quantitative IHC (for example, AQUA) and image analysis could further standardize delivery of multiplex panels that are far more cost-effective than complex molecular profiling. Rapid progress in image analysis and quantitative IHC [18,19] for other prognostic markers (ER, progesterone receptor, HER2, and so forth) suggests that this is an area of significant potential. One of the key requirements for any diagnostic pathology assay is that the assay is portable or reproducible across multiple centers. A key future step in the validation of the Mammostrat^® ^assay, therefore, is a demonstration that identical results can be derived on the same samples in different laboratories. Such ring studies have proven of significant value in validating other novel diagnostics, particularly in the field of HER2 testing [20,21].

## Conclusions

The present study provides further data on the use of Mammostrat^® ^in predicting the prognosis of early-stage, ER-positive, and ER-negative breast tumors. Although numbers of ER-negative and untreated cases were small, the evidence is consistent with Mammostrat^® ^acting as a prognostic tool in all early breast cancers. When viewed in context with other published studies, the data on stratification of ER-positive breast cancers across five independent cohorts is clearly most robust. The Mammostrat^® ^markers are biologically independent of one another and measure aspects of physiology distinct from proliferation, HER2 status, and hormone receptor status already assessed by IHC assays that are standard of care. Collectively these data add support to a potential role for Mammostrat^® ^in management of early-stage breast cancer.

## Abbreviations

DRFS: distant recurrence-free survival; ER: estrogen receptor; IHC: immunohistochemistry; OS: overall (breast cancer specific) survival; RFS: relapse-free survival.

## Competing interests

DTR, RSS, BZR and RAB are employees of and stockholders in Clarient Inc., which has filed patents on the Mammostrat^® ^assay.

## Authors' contributions

JMSB, JT, DTR, RSS and BZR contributed to the design of the study. FMC, LJ, JT and JMSB were responsible for the construction of tissue microarrays. GRK, WJ, IHK, UC, DAC were responsible for the clinical follow-up of patients. HCP, RAB and DTR performed and analyzed immunohistochemical staining. JMSB, AM, and GRK designed the statistical analysis plan and performed statistical analysis. JMSB, JT, AM, DTR and DAC helped write the manuscript. All authors approved the final version.

## Supplementary Material

Additional file 1 **Recurrence-free survival and overall survival for breast-conserving surgery breast cancers**. Figure S1 shows Kaplan-Meier recurrence-free survival curves for breast-conserving surgery breast cancers. Solid lines, Mammostrat^® ^score low risk; dotted lines, Mammostrat^® ^score medium risk; grey lines, Mammostrat^® ^score high risk. (S1A) Estrogen receptor (ER)-positive, node-negative breast cancers treated with adjuvant tamoxifen only. (S1B) ER-positive, tamoxifen-treated breast cancers (any nodal status). (S1C) All ER-positive breast cancers. (S1D) All breast cancers regardless of treatment and hormonal status. (S1E) Untreated breast cancers. (S1F) ER-negative breast cancers. Figure S2 shows Kaplan-Meier overall survival curves for breast-conserving surgery breast cancers. Solid lines, Mammostrat^® ^score low risk; dotted lines, Mammostrat^® ^score medium risk; grey lines, Mammostrat^® ^score high risk. (S2A) Estrogen receptor (ER)-positive, node-negative breast cancers treated with adjuvant tamoxifen only. (S2B) ER-positive, tamoxifen-treated breast cancers (any nodal status). (S2C) All ER-positive breast cancers. (S2D) All breast cancers regardless of treatment and hormonal status. (S2E) Untreated breast cancers. (S2F) ER-negative breast cancers. Click here for file
