# Supplementary material for: Mammostrat® as a tool to stratify breast cancer patients at risk of recurrence during endocrine therapy
Source: Breast Cancer Res. 2010 Jul 8;12(4):R47. doi: 10.1186/bcr2604 (PMC2949634; doi:10.1186/bcr2604)

Supplementary Figure 1A: RFS ER+ve/N-ve/Tam treated breast cancers.


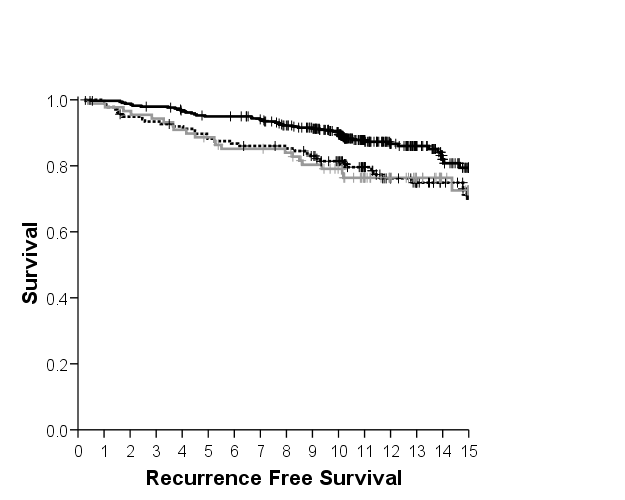


Supplementary 1B: RFS ER+ve/Nany/Tam treated breast cancers


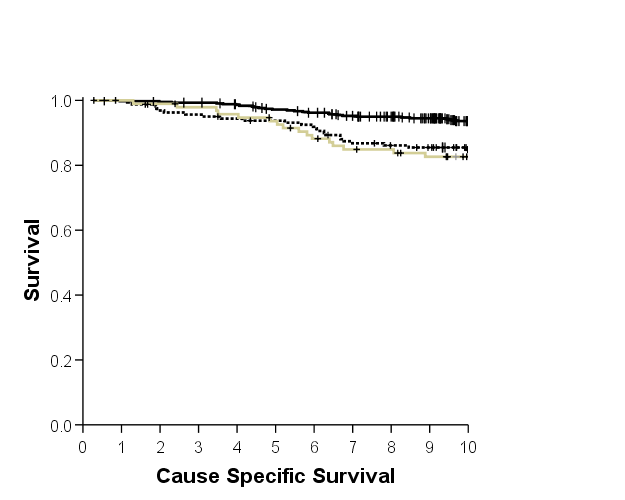


Supplementary Figure 1C: RFS All ER+ve breast cancers


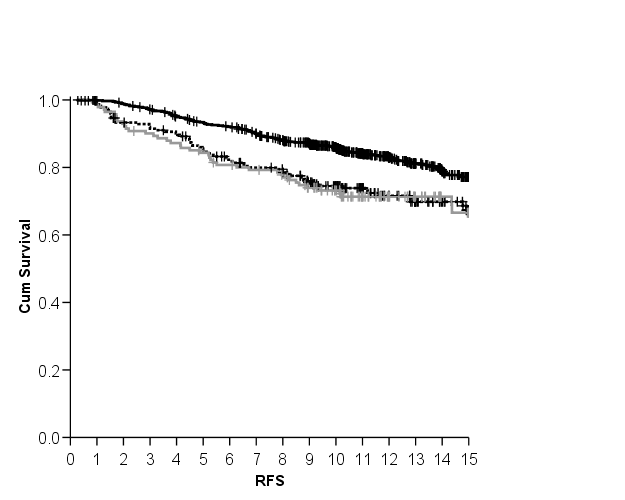


Supplementary Figure 1D:RFS All Cases:


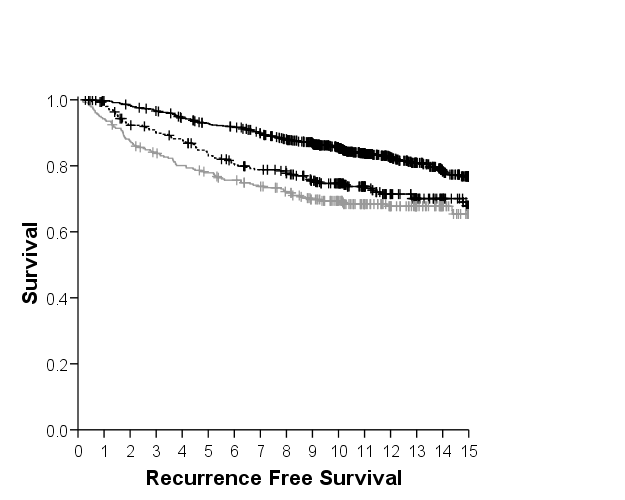


Supplementary Fig 1E: ER-ve cases RFS

Supplementary Fig 1F: Untreated cases RFS


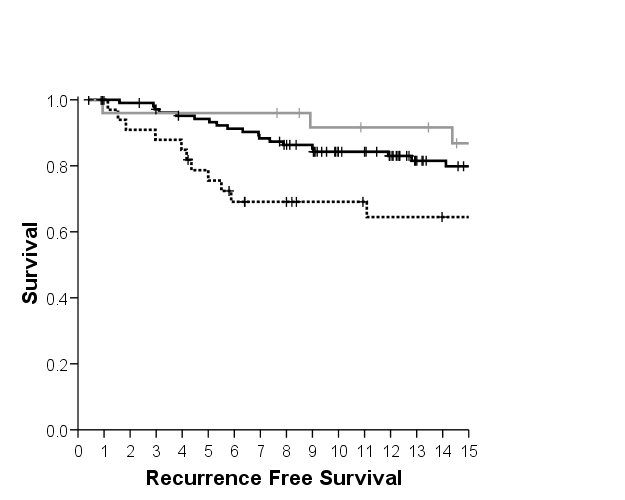


Supplementary Figure 2A: OS ER+ve/N-ve/Tam treated breast cancers

Data not shown

Figure 2B: OS ER+ve/Nany/Tam treated breast cancers


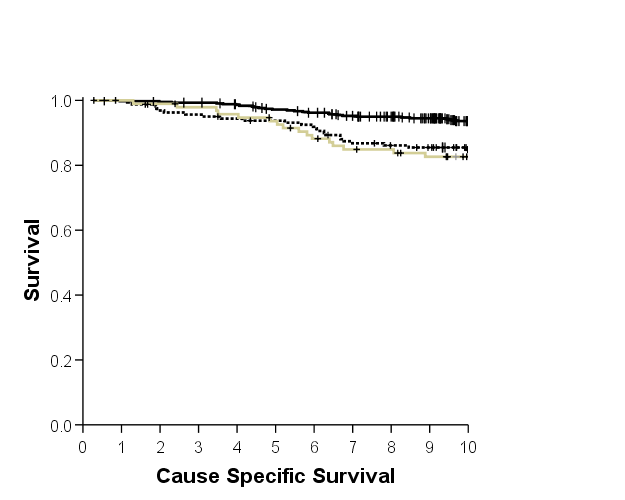


Supplementary Figure 2C: OS All ER+ve breast cancers


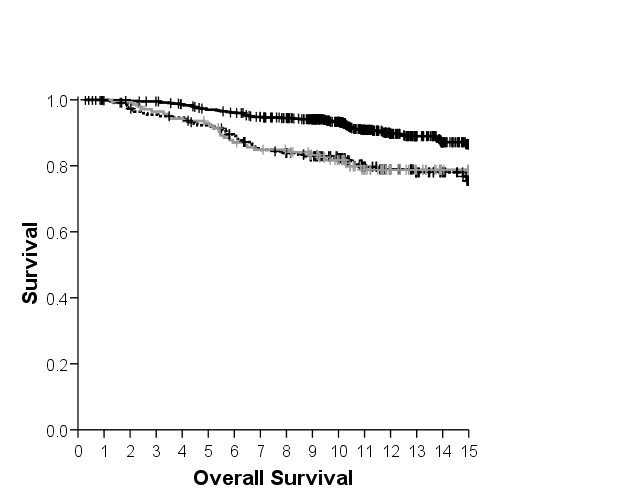


Supplementary Figure 2D: OS All cases


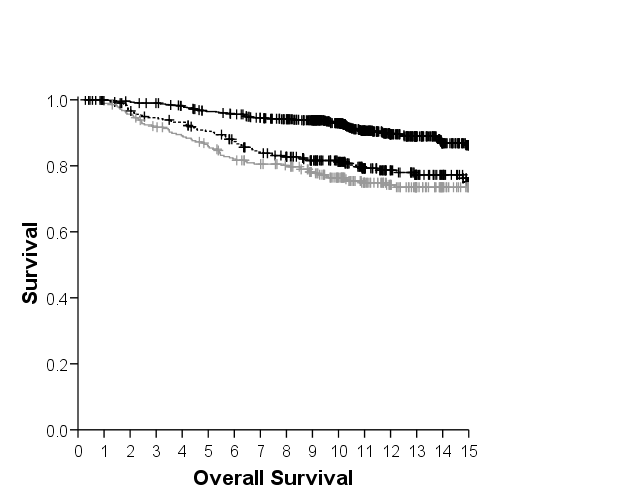


Supplementary Fig 2E: ER-ve cases overall survival __

Supplementary Fig 2F: Untreated Cases OS


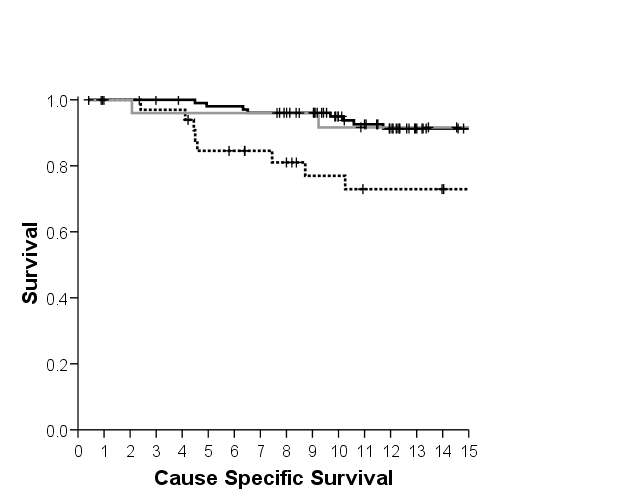

Supplement: Additional file 1 — Recurrence-free survival and overall survival for breast-conserving surgery breast cancers. Figure S1 shows Kaplan-Meier recurrence-free survival curves for breast-conserving surgery breast cancers. Solid lines, Mammostrat® score low risk; dotted lines, Mammostrat® score medium risk; grey lines, Mammostrat® score high risk. (S1A) Estrogen receptor (ER)-positive, node-negative breast cancers treated with adjuvant tamoxifen only. (S1B) ER-positive, tamoxifen-treated breast cancers (any nodal status). (S1C) All ER-positive breast cancers. (S1D) All breast cancers regardless of treatment and hormonal status. (S1E) Untreated breast cancers. (S1F) ER-negative breast cancers. Figure S2 shows Kaplan-Meier overall survival curves for breast-conserving surgery breast cancers. Solid lines, Mammostrat® score low risk; dotted lines, Mammostrat® score medium risk; grey lines, Mammostrat® score high risk. (S2A) Estrogen receptor (ER)-positive, node-negative breast cancers treated with adjuvant tamoxifen only. (S2B) ER-positive, tamoxifen-treated breast cancers (any nodal status). (S2C) All ER-positive breast cancers. (S2D) All breast cancers regardless of treatment and hormonal status. (S2E) Untreated breast cancers. (S2F) ER-negative breast cancers. [file bcr2604-S1.DOC]
